# Breast masses in mammography classification with local contour features

**DOI:** 10.1186/s12938-017-0332-0

**Published:** 2017-04-14

**Authors:** Haixia Li, Xianjing Meng, Tingwen Wang, Yuchun Tang, Yilong Yin

**Affiliations:** 1 School of Computer Science and Technology, Shandong University, Jinan, 250101 China; 2 School of Information, Shandong University of Political Science and Law, Jinan, 250014 China; 3 School of Computer Science and Technology, Shandong University of Finance and Economics, Jinan, 250014 China; 4 Research Center for Sectional and Imaging Anatomy, Shandong University School of Medicine, Jinan, 250012 China

**Keywords:** Breast mass, 1D signature contour subsection, RMS slope

## Abstract

**Background:**

Mammography is one of the most popular tools for early detection of breast cancer. Contour of breast mass in mammography is very important information to distinguish benign and malignant mass. Contour of benign mass is smooth and round or oval, while malignant mass has irregular shape and spiculated contour. Several studies have shown that 1D signature translated from 2D contour can describe the contour features well.

**Methods:**

In this paper, we propose a new method to translate 2D contour of breast mass in mammography into 1D signature. The method can describe not only the contour features but also the regularity of breast mass. Then we segment the whole 1D signature into different subsections. We extract four local features including a new contour descriptor from the subsections. The new contour descriptor is root mean square (RMS) slope. It can describe the roughness of the contour. KNN, SVM and ANN classifier are used to classify benign breast mass and malignant mass.

**Results:**

The proposed method is tested on a set with 323 contours including 143 benign masses and 180 malignant ones from digital database of screening mammography (DDSM). The best accuracy of classification is 99.66% using the feature of root mean square slope with SVM classifier.

**Conclusion:**

The performance of the proposed method is better than traditional method. In addition, RMS slope is an effective feature comparable to most of the existing features.

**Electronic supplementary material:**

The online version of this article (doi:10.1186/s12938-017-0332-0) contains supplementary material, which is available to authorized users.

## Background

**Fig. 1:**
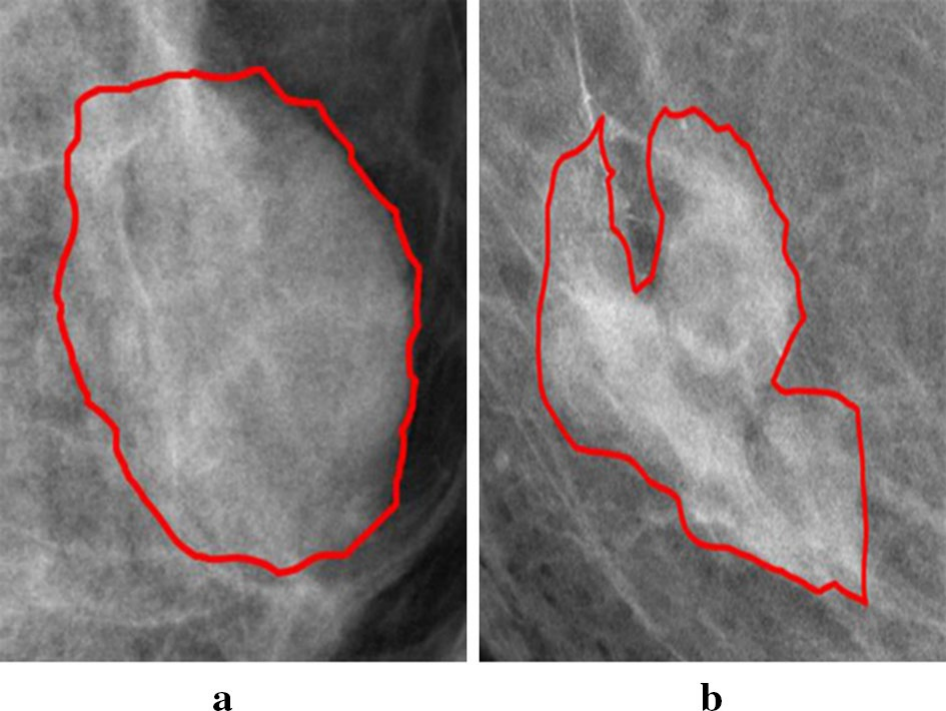
The breast mass in mammogram. **a** Benign mass with smooth margin and regular shape; **b** malignant mass with microlobulated margin and irregular shape

Breast cancer is now the most common cancer in women worldwide. Cases with 12.2% of all newly diagnosed breast cancers and 9.6% of all deaths from breast cancer are contributed by China [1]. Early detection of breast cancer can increase survival rate [2]. Currently, mammography is the most reliable method for detection of the abnormality in the breast [3–5]. But it is still a challenging work for the radiologists to distinguish between the malign and benign mass. Abnormal cases have various contour shapes, textures, and sizes. It is very difficult even for experienced radiologists to discriminate whether the breast mass is malign. Now the diagnoses depend on biopsy puncture which brings hurts to mind and body of the patients. A computer-assisted-diagnose system, which merges image processing and pattern recognition theory, can provide the diagnosis suggestions to decrease the false detection rate and false negative rate [6]. In screening mammography, if a doctor sees a clearly defined mass whose contour is microlobulated or spiculated, he need not ask patient to do pathological puncture. He is quite sure that the mass is malignant Fig. 1b. If the contour of a breast mass is regular and the shape is nearly round, then the mass is probably benign Fig. 1a. The computer assisted diagnose can distinguish the two classes breast mass. It can decrease the pain of patient to do pathological puncture.

Researchers proposed many methods to describe the shape and texture in the system of CAD. Shape descriptor is compactness, eccentricity, moment, Fourier transformation descriptor, statistical marginal characteristics [7–11]. Texture descriptions gray level co-occurrence matrix and fractal dimension and so on [5, 12–14]. Pohlman et al. [15] proposed a method to transform 2D contour of breast mass to 1D signature. The signature of a contour is obtained by a function of radial distance from the centroid to the contour versus the angle of the radial line over the range (0°–360°). In this way, a signature of small fluctuation is obtained if the contour of breast mass is benign. Otherwise, if it is a malignant mass, a signature of large fluctuation is obtained. Fractal character can describe the fluctuation. So in literature [16] the breast mass is classified with the fractal analysis and the classification accuracy is greater than 80%. However, the function of radial versus degree could lead to a multi-value function in the case of an irregular or speculated margin [17]; the signature computed in this manner would also have ranges of undefined values in the case of a contour for which the centroid falls outside the region enclosed by the contour. Rangaraj et al. [16] improved the method. They transformed the 2D contour of breast mass to 1D signature by polygonal modeling of contours of breast masses using the turning angle function. Rangayyan and Nguyen [2] demonstrated the usefulness of fractal analysis for the classification of breast masses with the box-counting and ruler methods for the derivation of the FD of the two-dimensional 2D contours of masses as well as their one-dimensional 1D signatures. Some literatures [2, 9, 18–20] revealed that the regular extent is also very important to make a distinction between benign and malign breast mass. If the shape of mass is circular or oval then its probability to be benign is larger than to be malign mass.

**Fig. 2:**
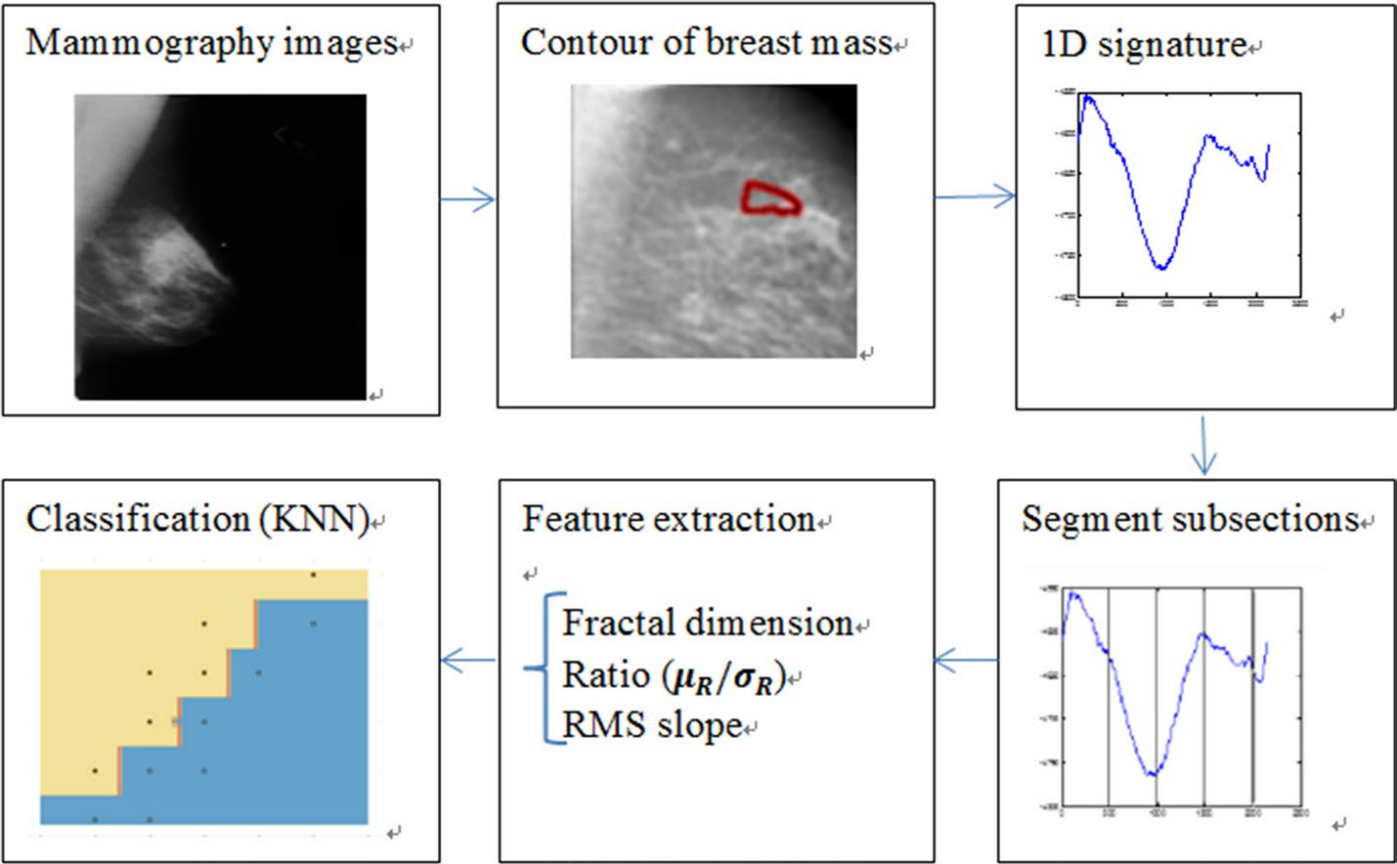
Flowchart of our proposed method

So we propose a new method in this paper to express the regularity of the contour for breast mass. At first, abnormal area in the mammography image is labelled by experimental doctors. Second, we translate 2D contour to 1D signature using the Euclid distance from the edge of the breast mass to periphery of the circular or oval centered with centroid. This method describes not only the roughness of the contour but also the regular degree of the contour. Third, we segment the whole 1D signature into different subsections. Fourth, we extract several local contour features. At last, the feature vectors re-organized according to the local feature value of each subsection are fed different classifiers. The flowchart of our proposed is shown as Fig. 2.

The remainder of this paper is organized as follows. The new method for translating 2D contour to 1D signature is proposed in “Methods”. In “Features”, we extract fractal dimension FD, $w$, $\mu_{R} /\sigma_{R}$ (where $\mu_{R}$ means mean radial distance of tumor boundary, and $\sigma_{R}$ means standard deviation), and root mean square slope features describing the contour characteristic. Then in the next Section, experimental results and analysis are introduced. The last is the summary of our work and the prospect of future work.

## Methods

In this part, the database is firstly introduced. Second, the method of 2D contour to 1D signature is illustrated in some detail. Finally, we explain how to segment 1D signature into subsections and how to re-organize these subsections.

### Database

In this paper, digital database for screening mammography (DDSM) has been utilized to provide the mammography images. This database is provided by the Massachusetts General Hospital, the University of South Florida, and Sandia National Laboratories [21, 22]. This database includes about 2620 cases. Each case has 4 mammography images composed of two view images of each breast, along with some associated patient information. Images containing suspicious areas have associated pixel-level ground truth information about the locations and types of suspicious regions. This information is saved as an overlay file. Each overlay file may specify multiple abnormalities. Each abnormality has information on the lesion type, the assessment, the subtlety, the pathology and at least one outline. Each boundary is specified as a chain code. The details about the DDSM database can be found in literature [23] or availability of data and materials at the end of this article. The database includes Normal, benign and cancer volumes. The research object in this article is the contour of benign and malignant mass. So we choose 323 contours of mammography images from DDSM database including 143 contours of benign images and 180 contours of malign images. In order to simplicity and convenience of experiment, we choose some mammography images including single abnormality. The numbers of the images of we used are listed on the Additional file 1: Appendix S1. Among 143 benign images, most contours are similar ellipse. These benign mass is prone to classify wrongly using existing method. All images are from the different patient.

## 2D contour to 1D signature

**Fig. 3:**
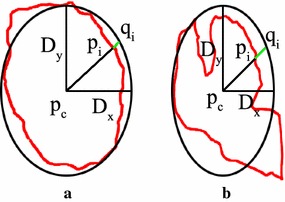
Contours of breast mass. **a** Benign mass; **b** malignant mass

**Fig. 4:**
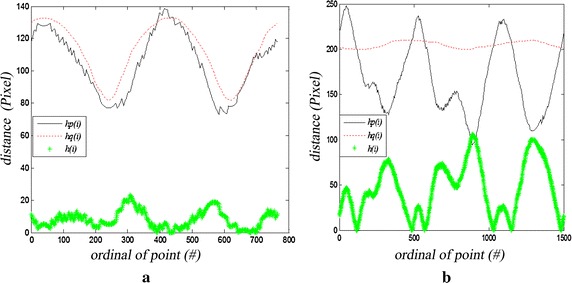
1D signatures of breast mass contours. **a** Benign mass; **b** malignant mass

The benign mass has a smooth shape that results in a simple signature, whereas the malignant tumor has a jagged contour that leads to a rough signature. The contours of every abnormality are extracted by means of connecting the point expressed with chain code in the overlay files. Figure 3a and b show the contours of benign breast mass and malignant mass. The contour of a 2D contour can be formalized as an orderly point set along anticlockwise direction $C = \{ p_{i} = (x_{i} ,y_{i} ),i = 1,2,3, \ldots ,N\}$. $(x_{i} ,y_{i} )$ is the coordinate of point $p_{i}$ and N is the number of point on the contour restricted by $p_{i + N} = p_{i}$. The center $p_{c} (x_{0} ,y_{0} )$ of 2D contour is expressed as $(x_{0} ,y_{0} ) = \left( {\tfrac{{\sum\nolimits_{i = 1}^{N} \,x_{i} }}{N},\tfrac{{\sum\nolimits_{i = 1}^{N} \,y_{i} }}{N}} \right)$. The first point on the contour we choose is on the right of center point. It is the crossover point of the horizontal line passed through the center point and the contour of breast mass. Radius is the distance between the point $p_{i}$ on the contour and the center $p_{c} (x_{0} ,y_{0} )$. The diameter of one axis $x$ is $D_{x} = \mathop {\text{max}}\nolimits_{{i,j \in \{ 1, \ldots ,N\} }} |x_{i} - x_{j} |$. The diameter of the other axis $y$ is $D_{y} = \mathop {\text{max}}\nolimits_{{i,j \in \{ 1, \ldots ,N\} }} |y_{i} - y_{j} |$. So the equation of the ellipse centered as $p_{c} (x_{0} ,y_{0} )$ and diameter as $D_{x} ,D_{y}$ respectively is $\tfrac{{x - x_{0} }}{{D_{x} }} + \tfrac{{y - y_{0} }}{{D_{y} }} = 1$. If $D_{x} = D_{y}$, the ellipse is transformed into a circle centered as $p_{c} (x_{0} ,y_{0} )$ and diameter is $D_{x} (D_{y} )$. This ellipse or circle is the standard of breast mass contour. If the points on the contour of breast mass are all near the ellipse, we can declare the contour is regular. The probability that the mass is benign is high. Otherwise, the mass is determined as malign. We define that $h_{p} (i)$ is the distance function between $p_{c} (x_{0} ,y_{0} )$ and $p_{i} (x_{i} ,y_{i} )$. $h_{q} (i)$ is the distance function between $p_{c} (x_{0} ,y_{0} )$ and $q_{i} (x_{i} ,y_{i} )$. The distance between $p_{i} (x_{i} ,y_{i} )$ and $q_{i} (x_{i} ,y_{i} )$ is defined as $h(i)$. $h$ is also the function of the number of pixel on contour. Figure 4a and b show 1D signature of benign and malign breast mass in Fig. 3a and b.

### Subsection and integration

The method which 2D contour transforms into 1D signature can describe the feature of the whole contour. Sometimes the local feature is also very important to classify the benign and malignant breast mass. In Fig. 1b, for example, the $2/3$ contour in the left is smooth and regular but subsection in the right is microlobulated. It is not precise if we extract the feature on whole contour. So we propose a method that a whole signature is divided into $C \in \{ 1,\;2,\;4,\;6,\;8,\;10,\;12,\;14,\;16,\;18,\;20\}$ subsections respectively. If C = 1, the signature is whole one. The feature is a value. Otherwise, the feature of each subsection is extracted respectively. Then segments are ranked by the value of each subsection feature. Finally these subsections are integrated into a whole signature in sequential order according to the value of feature. That is to say the feature of each contour of breast mass is a vector of C dimension. The number of subsections affects the accuracy of classification. Because the optimized amounts of subsections are relevant to the size of mass contour and features, we divide each contour into C subsections and choose the average accuracy of all kinds of subsections as the final performance of each feature. For example, if C = 4, each contour is segmented into 4 subsections. The feature is a vector of 4 dimensions. Then we feed 323 feature vectors into classifiers. After the whole set C is ergodic, we obtain 11 results. The average of 11 results is as the final performance.

## Features

In this part, four features are introduced. Among them, RMS $s$ is first proposed by us. It can describe the variation of 1D signature in vertical direction well.

### Root mean square roughness w

Root mean square roughness describes the irregular degree of 1D signature. The root mean square roughness is defined as: $w$ is root mean square roughness defined as $w = \sqrt { \langle h^{2}\rangle  - \langle h \rangle ^{2} }$. Among the equation, 〈 〉 expresses the statistical average, $w$ expresses the fluctuation degree of $h$ in vertical direction. The shape is more regular with the value of more small. That is to say that the margin is more close to a circle or ellipse. The mass will more probably be benign than malign. So root mean square roughness may be used as a feature to classify the benign or malign breast mass.

### The $\mu_{R} /\sigma_{R}$ ratio

The $\mu_{R} /\sigma_{R}$ ratio (where $\mu_{R}$ means mean radial distance of tumor boundary, and $\sigma_{R}$ means standard deviation), describes the circularity of the breast mass contour. Malignant mass should have smaller values of circularity than benign mass. Haralick [24] proved that the $\mu_{R} /\sigma_{R}$ ratio is a good feature in classifying malignant mass and benign mass. Polhman [15] applied this feature in his 1D signature and acquired the good result.

### Fractal dimension

According to the fractal geometry of Mandelbort, the fractal dimension can describe the property of self-similarity in some way. Many fractal models are proposed to analyze fractal phenomenon of nature. The popular fractal model is differential box-counting method. Studies prove that the differential box-counting method is appropriate to self-similarity fractal model. In medical image, the fractal Brownian motion (fBm) model has been shown to be suitable for the analysis of medical image because the intensity surface of a medical image can be viewed as the end result of random walk. The fBm model belongs to the class of statistically self-affine fractal concept and regards naturally occurring rough surfaces as the end result of random walks. Since the roughness of the intensity surface of a medical image can also be viewed as the end result of a random walk, the fBm model suits for the analysis of medical images. To the affine fractal random rough model, autocorrelation function and height-height correlation function can be expressed as [23]:

1 $$ R(\rho ) = w^{2} exp[( - \rho /\xi )^{2\alpha } ] $$

2 $$ H(\rho ) = \langle [h(n) - h(n^{\prime})]^{2} \rangle = 2w^{2} \{ 1 - exp[( - \rho /\xi )^{2\alpha } ]\} $$

where $\alpha$ is the fractal exponent, the relative between $\alpha$ and fractal dimension $D$ is $\alpha = d - D$, $d$ is the space dimension, and $\alpha$ is constraint by $0 \le \alpha \le 1$. $w$ is root mean square roughness expressing the fluctuation degree of $h$ in vertical direction, and $\xi$ is correlation length expressing the fluctuation degree of $\rho$ in horizontal direction. The autocorrelation function $R$ of $h(i)$ is can be defined as:

3 $$ R(i + \rho ) = \langle h(i)h(i + \rho ) \rangle /w^{2} $$

Here, $\rho = |i_{2} - i_{1} |$ is the interval between two points on signature. The autocorrelation function $R$ has some characteristics such as: (1) If the signal is the smooth and steady random process, $R(i + \rho )$ is irrelevant to $n$ and relevant to only $o$ i.e. $R(i + \rho ) = R(\rho )$. With the increment of correlation interval $\rho$, $R(\rho )$ decreases little by little and tends to be zero. The rate of decrease is decided by the distance between two points irrelevant to each other. The correlation length is defined by the value of correlation interval at the point that the autocorrelation function $R(\rho )$ decreases to $e^{ - 1}$ of the maximum. The correlation length $\xi$ expresses the speed that $R(\rho )$ decreasing with $\rho$.If the interval between two points is less than $\xi$, the two points are correlated. Otherwise, the two points are independent. The fluctuation in the horizontal direction is expressed with $\xi$ and the fluctuation in the vertical direction is expressed with $w$.

In the condition of $\rho < < \xi$, self-affine fractal surface $h(n)$ satisfies self-affine transform below:

4 $$ h(x_{0} ,y_{0} ) = \varepsilon^{2\alpha } h(\varepsilon x_{0} ,\varepsilon y_{0} ) $$

If the scale is small as $1/\varepsilon$, the average variation of height difference is $\varepsilon^{2\alpha }$. This variation is corresponding to the power law variation of height-height correlation function during the short distance. The relationship is

5 $$ h(\rho ) \propto \rho^{2\alpha } ,\rho < < 1 $$

**Fig. 5:**
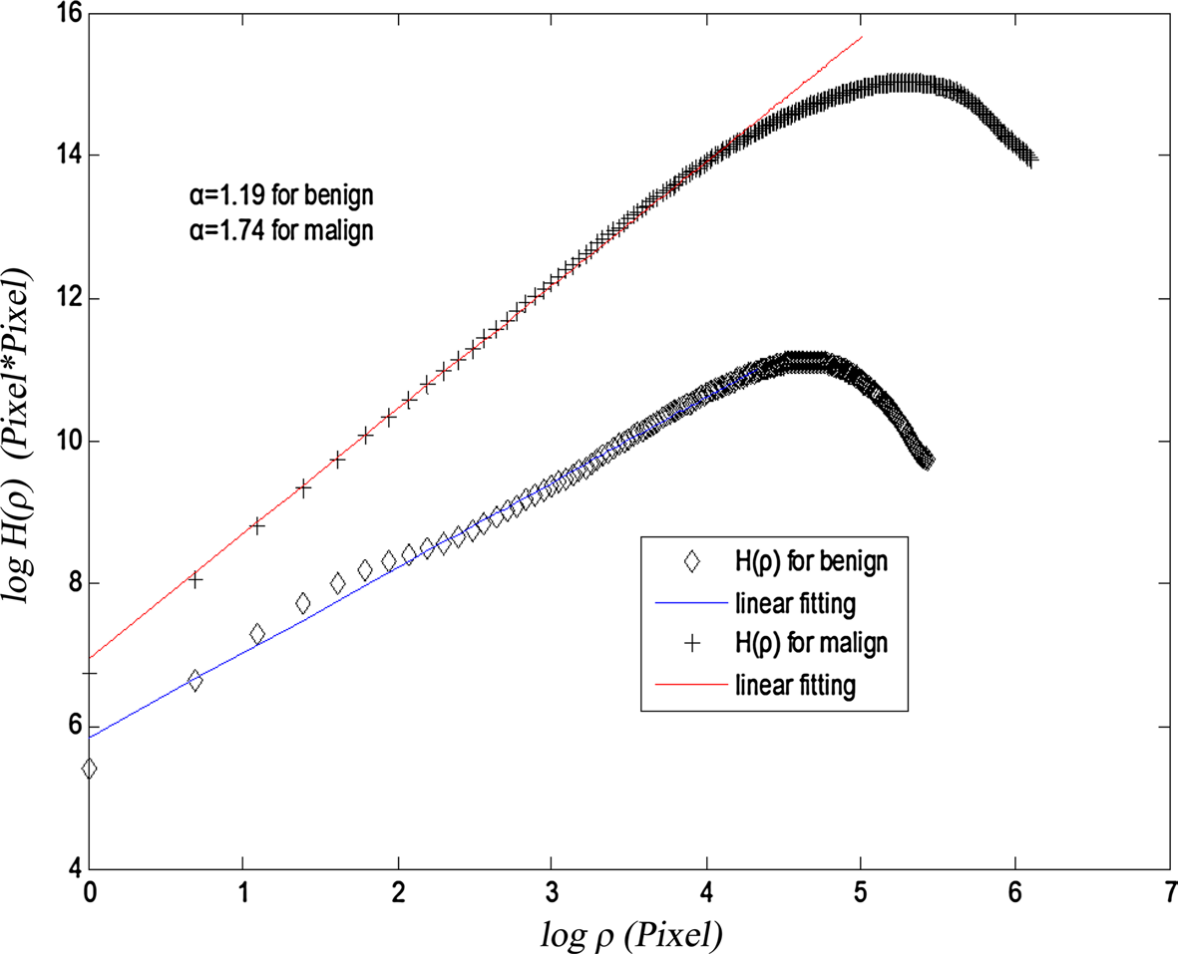
Height–height correlation function curve and linear fitting for benign and malign mass contour in log–log coordinate

The power law variation of height-height correlation function can describe statistically self-similarity characteristic and local fluctuation. If $\alpha$ is smaller, the local fluctuation is more violent and fractal dimension is larger. From the Eq. (5), we can conclude that in log–log coordinate system $h(\rho )$ is proportional to $\rho$ when $\rho < < 1$. $2\alpha$ can be estimated from the slope of the line approximated by linear least squares fitting on $log(H(\rho ))$ versus $log(\rho )$ when we choose a range of the lower scale $\rho$. Figure 5 shows the curve of $log(H(\rho ))$ versus $log(\rho )$ and the linear fitting for benign and malign mass. In this paper, we look the 1D signature of contour as height distribution of the affine fractal random surface. The fractal dimension indicates the self-similarity feature and it also expresses the local non-smooth fluctuation of the signature. The fractal dimension D is larger and larger; the local fluctuation of the signature is more and more drastic. Here we use the fractal exponent $\alpha$ of 1D signature of contour as the third feature to distinguish the benign mass from the malign one.

### RMS slope $s$

Each point on the contour has different slope. The variation of slope describes the shape of contour. If the contour is smooth, the variation of slope is slow and regular; otherwise, variation of slope is drastic. When we transform 2D contour into 1D signature, the value in the Y-axis expresses the circularity. The absolute value of the slope shows the variation speed of contour. So we take the slope distribution of each point on the contour as one of the features to discriminate malign mass from benign mass. Slope is acquired by linear interval. Root mean square slope is defined as:

6 $$ s = \sqrt { \left\langle \left (\frac{dh(\rho )}{d(\rho )}\right)^{2} \right\rangle } $$

We can see from the Fig. 4 and Eq. (6) that the slope of benign mass has small value and the fluctuation is gentle. While the slope of malignant mass has big value and the fluctuation is violent. The variation range of the RMS $s$ for malignant mass is wider than benign mass.

## Classification

K-Nearest-neighbor (KNN), support vector machine (SVM) and artificial neural network (ANN) are used as classifiers in this paper to differ benign mass from malign mass of breast. We choose K = 1 in KNN classifier and use a linear support vector machine classifier. The NNet classier is configured with 10 nodes in the hidden layer. The internal weight is initialized with randomly chosen values. 323 contours are divided into two subsets 300 contours for training and 23 for testing. The software we use is Matlab R2015b on a Win10 Operating System.

## Experimental results and analysis

In this part, the performance of the proposed method is reported. Then, performance of four features is compared. Third, the effect of subsections is analyzed. And finally, classifier performance is shown.

### Performance evaluation for 2D contour to 1D signature

**Fig. 6:**
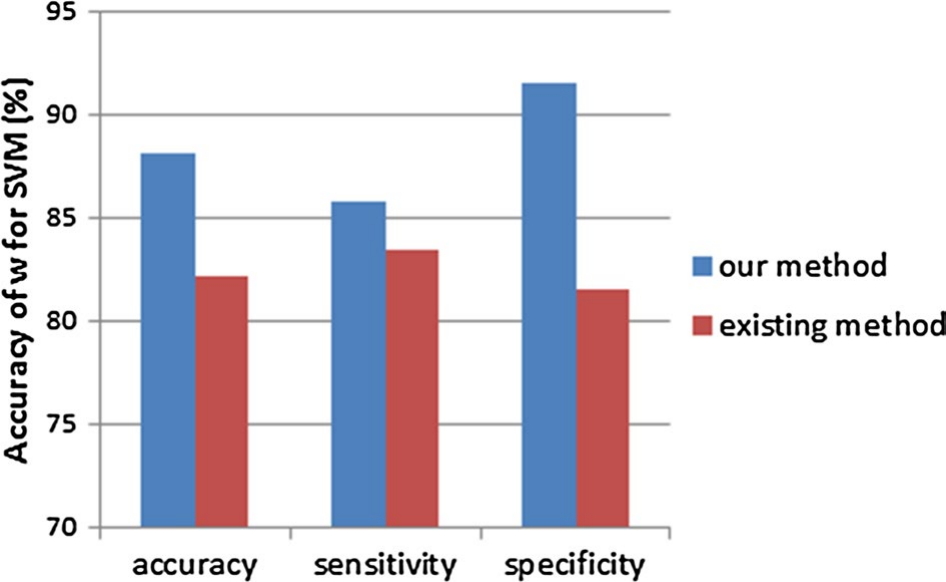
The bar chart of accuracy, sensitivity and specificity for our method compared with existing method used SVM classifier

Table 1 show the comparison of our proposed method and existing method. We can see that the accuracy used our method is higher than used existing method. The obvious promotion is the accuracy of alpha. It raises 14.90%, whereas the accuracy of RMS $s$ barely changes. This is because the accuracy of RMS $s$ itself is close to 100%. It is difficult to rise greatly. To similar ellipse cases of breast mass in selected database, our proposed method can not only describe the circularity of contour but also illustrate the degree of margin fluctuation. While traditional method used only the standard deviation of median filtering and origin boundary to quantify the degree of margin fluctuation. From Fig. 6 we can see that whether accuracy or sensitivity and specificity are improved with our method. Especially, the specificity of w for SVM raises 10.90%.

**Table 1:** The accuracy comparison of our work $h(i)$ with traditional one $h_{p} (i)$

Feature	Method	KNN (%)	SVM (%)	ANN (%)
$w$	$h_{p} (i)$	76.68	82.21	79.84
	$h(i)$	81.82	88.14	88.54
$\mu_{R} /\sigma_{R}$	$h_{p} (i)$	76.68	84.51	79.45
	$h(i)$	81.82	90.57	88.54
$\alpha$	$h_{p} (i)$	83.00	87.21	83.79
	$h(i)$	86.17	91.92	89.33
$s$	$h_{p} (i)$	92.09	99.33	94.86
	$h(i)$	92.47	*99.66*	99.60

Italic type indicate maximum value

### Performance evaluation for four features with three classifiers

**Fig. 7:**
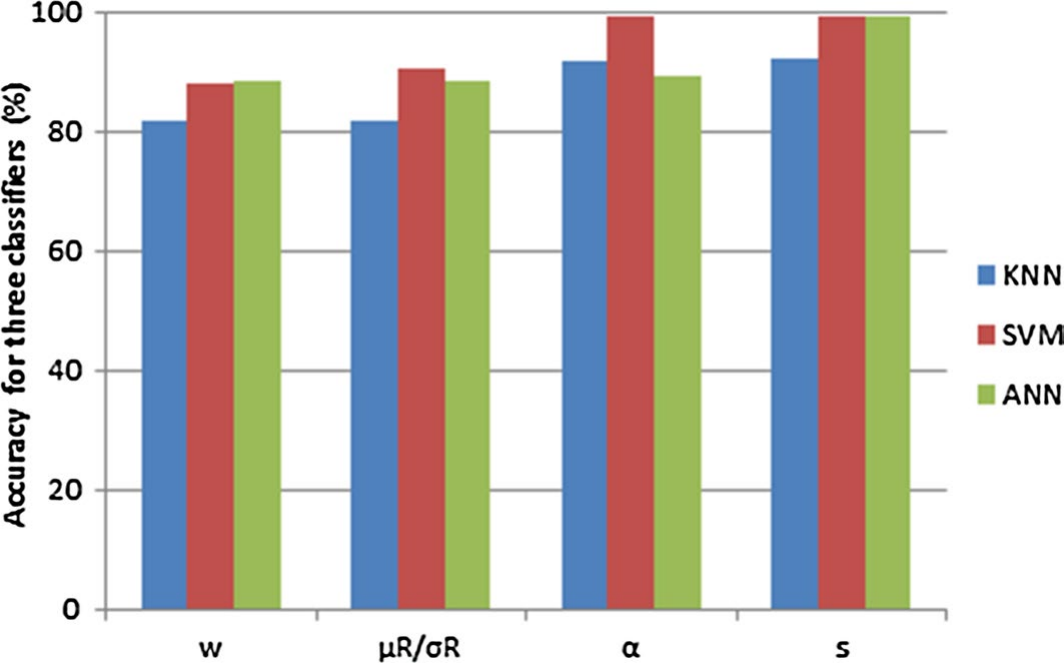
The bar chart of accuracy for four features used KNN, SVM and ANN classifier

Figure 7 and Table 1 show the performance of four features with three different classifiers. No matter which classifier is used, the result proves that our proposed feature is better than existing one. To the features $w$ and $s$, SVM classifier is the almost the same as ANN and is better than KNN. To other features, SVM is the best among these three classifiers. SVM is robust for small sample data. The accuracy of fractal feature $\alpha$ is 99.33%. Its performance is better than $w$ and $\mu_{R} /\sigma_{R}$. This is because the 1D signature of contour for breast mass accords with the fractal characteristic. The highest accuracy is 99.96% using the feature of root mean square slope with SVM classifier. The reason is that RMS slope can describe the variation of vertical direction of 1D signature. It is very important to distinguish the benign mass and malignant one.

### Performance evaluation for subsection

**Fig. 8:**
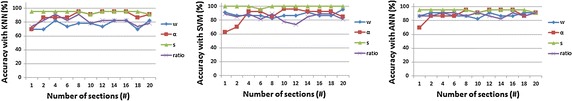
Curve of sections V.S. accuracy for four features

Figure 8 shows the performance of four features for subsection using $h(i)$ proposed in this paper. Performance is improved due to considering the local features in our method. Experiment proves that subsection is efficient to improve the performance for four features. Due to the slope feature has high performance, the improvement is not obvious. It can be seen that the accuracy increases quickly with the increasing the number of the subsections at the start for the feature of fractal dimension. Later the performance is stable with the larger N. This is because when N is larger, the segment is shorter; the number of point on the contour is less. The accuracy is affected due to the less point on the subsection. In three classifiers, SVM acquire the best performance using the feature of RMS slope. The performance of subsection is stable using the ANN classifier for four features.

## Conclusion and future work

It is very important for contour to distinguish the benign breast mass from malign one. In this paper, we propose three shape features of broken line for contour to classify the benign and malign breast mass. The accuracy rate attains 99.66% with the RMS slope feature. In addition, we compute fractal dimension by another method of height-height correlation function in log–log coordinate. The accuracy rate attains 99.33%. It is higher than $\mu_{R} /\sigma_{R}$ and $w$. For further researches, the selection of N and some texture features could be studied for improving the classification performances. We can choose more cases in order that our study has a wider application range. Also, more advanced classification methods such as deep neural network can be used to improve the classification accuracy.

## Additional file

**Additional file 1: Appendix S1.** List of the file names we used in the experiment. This file can be opened in the Microsoft Excel 2010.

## Abbreviations

RMS: root mean square
KNN: K-nearest neighbor
SVM: support vector machine
ANN: artificial neural network
DDSM: digital database of screening mammography
FD: fractal dimension
ROI: region of interest
